# A multimodal deep learning framework for clinical nursing assessment in lumbar fusion surgery via representation learning and feature extraction

**DOI:** 10.1038/s41598-026-51495-x

**Published:** 2026-05-07

**Authors:** Chao Li, Jianshu Cai, Xiaoling Huang, Yan Ni, Xuping Ling, Shibiao Fang

**Affiliations:** 1 Nursing Department, Sir Run Run Shaw Hospital, Zhejiang University School of Medicine, Hangzhou, 310000 Zhejiang Province China; 2 Zhejiang University, Hangzhou, 310000 Zhejiang Province China

**Keywords:** Multimodal learning, Lumbar spine imaging, Medical image analysis, Image–text alignment, Cross-modal representation, Lumbar fusion surgery, Computational biology and bioinformatics, Health care, Mathematics and computing, Medical research

## Abstract

Medical image interpretation plays a critical role in lumbar fusion surgery, where accurate analysis of anatomical structures is essential for clinical assessment. However, most existing deep learning approaches rely primarily on visual features and fail to effectively integrate heterogeneous clinical information. This study proposes a multimodal deep learning framework for lumbar spine image interpretation by jointly modeling medical images and associated clinical text. The framework adopts a global–local representation learning strategy to capture both overall anatomical context and fine-grained structural information. A visual encoder extracts hierarchical features from lumbar radiographs and CT scans, while a transformer-based text encoder captures semantic information from clinical reports. These representations are projected into a shared embedding space to enable cross-modal alignment. To enhance feature interaction, a text-guided attention mechanism is introduced to model correspondence between image regions and textual descriptions. The learned multimodal representations are applied to multiple downstream tasks, including cross-modal retrieval, classification, and lumbar structure segmentation. Experimental results show that the proposed framework outperforms image-only baselines and achieves competitive performance compared with existing multimodal approaches. The integration of global and local representations improves feature discrimination and structural modeling. Visualization results provide qualitative evidence that the model focuses on anatomically relevant regions, although such observations should be interpreted with caution. Overall, the proposed framework demonstrates the potential of multimodal representation learning for lumbar spine image analysis and provides a structured approach for integrating heterogeneous clinical data.

**Supplementary Information:**

The online version contains supplementary material available at 10.1038/s41598-026-51495-x.

## Introduction

 Medical image analysis plays a critical role in clinical diagnosis, treatment planning, and postoperative evaluation, particularly for musculoskeletal disorders. Degenerative lumbar spine diseases are among the leading causes of chronic low back pain and disability, and lumbar fusion surgery is widely performed to restore spinal stability and relieve neurological symptoms^1–4^. However, accurate interpretation of lumbar spine imaging remains challenging due to complex anatomical structures, inter-patient variability, and the presence of postoperative instrumentation.

Recent advances in deep learning have significantly improved medical image analysis, especially in tasks such as segmentation and classification. Traditional single-modality approaches, including CT- and ultrasound-based methods, have achieved notable progress in extracting structural information^5–10^. Nevertheless, these methods primarily rely on visual features and often suffer from limited robustness and insufficient clinical interpretability in complex scenarios. Even with modern convolutional neural networks such as U-Net, most existing approaches remain constrained by single-modality inputs^11–15^. In clinical practice, however, medical image interpretation is inherently multimodal. Radiological images are typically accompanied by textual clinical records, including diagnostic reports, surgical notes, and postoperative assessments^16–20^. These textual descriptions contain rich semantic information that complements visual features. Therefore, integrating visual and textual information has become an important direction for improving both representation quality and clinical relevance. Recent multimodal learning approaches have demonstrated the effectiveness of aligning visual and textual data within a shared embedding space, enabling tasks such as cross-modal retrieval and classification^21–25^. However, most existing methods are developed for natural image domains and are not directly applicable to medical imaging scenarios. In particular, lumbar spine imaging requires precise modeling of structural consistency and subtle anatomical variations, as well as fine-grained correspondence between anatomical regions and clinical concepts.

Moreover, recent studies have emphasized that multimodal medical AI systems benefit from structured multi-stage design, where representation learning, feature extraction, and downstream prediction are integrated within a unified framework^26^. At the same time, hybrid modeling approaches involving multiple components require rigorous ablation analysis and careful interpretation of results to ensure reliability and avoid overestimation of model capabilities^27^.

Despite these advances, a clear gap remains in developing unified multimodal frameworks that can simultaneously achieve: (1) fine-grained anatomical–semantic alignment, (2) robust representation learning across heterogeneous data, and (3) consistent performance across multiple downstream tasks.

To address these challenges, this study proposes a multimodal deep learning framework for lumbar spine image interpretation based on global–local representation learning. The proposed method integrates visual features from medical images with semantic representations derived from clinical text into a unified embedding space. Specifically, global representations capture overall anatomical context and clinical semantics, while local representations model fine-grained correspondences between image regions and clinically relevant terms. In addition, a text-guided attention mechanism is introduced to enhance cross-modal interaction by highlighting relevant anatomical regions. Based on the learned multimodal representations, the framework supports multiple downstream tasks, including cross-modal retrieval, classification, and anatomical structure segmentation.

## Method

Following the problem formulation described in the Introduction, this section presents the proposed multimodal framework for integrating visual and textual information in lumbar spine analysis.

### Multimodal feature encoding for lumbar fusion imaging

In this study, we consider paired multimodal inputs consisting of a medical image $\:{\mathrm{x}}_{\mathrm{v}}$ and its corresponding clinical description $\:{\mathrm{x}}_{\mathrm{t}}$. To effectively capture complementary information from both modalities, we employ two dedicated encoders: a visual encoder $\:{E}_{\mathrm{v}}$and a textual encoder $\:{E}_{\mathrm{t}}$. The visual encoder extracts hierarchical representations from spinal images, including both holistic anatomical context and localized structural details such as vertebral alignment, implant positioning, and intervertebral spaces. Simultaneously, the textual encoder processes clinical narratives to generate semantic embeddings at both the document level and the token level, capturing key medical concepts relevant to lumbar fusion procedures.

Specifically, we decompose the learned features into two categories: (1) global representations, which summarize the overall condition of the lumbar region and surgical outcomes, and (2) local representations, which focus on fine-grained correspondences between image sub-regions (e.g., specific vertebrae or fusion sites) and clinically meaningful terms (e.g., “fixation”, “degeneration”, “fusion segment”).

These multimodal features are jointly optimized within a unified representation learning framework, enabling the model to align visual patterns with textual semantics. The resulting representations are subsequently leveraged for downstream tasks, including automated interpretation, classification of surgical outcomes, and precise segmentation of anatomical structures relevant to lumbar fusion.

#### Visual feature encoding

To model the visual modality, we employ a convolutional neural network as the backbone to extract hierarchical representations from lumbar spine images. Specifically, a ResNet-based architecture is adopted as the visual encoder $\:{E}_{\mathrm{v}}$, due to its strong capability in capturing structural and anatomical patterns in medical imaging. The encoder produces two levels of visual representations. The global feature vector $\:{\mathrm{f}}_{\mathrm{g}}\in{\mathrm{R}}^{\mathrm{C}}$ is obtained from the final pooling layer, summarizing the overall anatomical configuration of the lumbar region, including vertebral alignment, surgical instrumentation, and global pathological conditions. In addition to global representations, we extract localized visual features from intermediate convolutional feature maps. These feature maps are spatially partitioned into ***M*** regions, and each region is encoded into a *C*-dimensional feature vector, resulting in local representations $\:f_{l} \in R^{{C \times M}}$. These local features enable the model to capture fine-grained structural variations, such as specific vertebral segments, fusion sites, and implant-related details, which are critical for precise interpretation and segmentation in lumbar fusion surgery.

#### Clinical text encoding

Clinical reports associated with lumbar fusion procedures often contain complex descriptions spanning multiple sentences, including diagnostic findings, surgical details, and postoperative evaluations. To effectively capture such long-range semantic dependencies, we utilize a transformer-based language model as the text encoder $\:{E}_{\mathrm{t}}$. In particular, a domain-adapted pretrained model of BioClinicalBERT is employed to encode medical narratives into contextualized embeddings. This enables the extraction of clinically meaningful semantic representations aligned with spinal imaging features. To address the prevalence of abbreviations, domain-specific terminology, and inconsistencies in clinical documentation, we adopt a subword tokenization strategy. Given a clinical report consisting of ***W*** words, each word is decomposed into multiple subword units, yielding a sequence of ***N*** tokens. These tokens are then fed into the text encoder to generate contextualized embeddings. The encoder outputs local textual representations $\:g_{l} \in R^{{K \times N}}$, where each token is represented by a *K*-dimensional feature vector. To obtain a holistic understanding of the report, we further aggregate these token-level features into a global textual representation *g*_*g*_, which summarizes the overall clinical semantics of the report. This dual-level textual representation enables the model to align fine-grained clinical concepts (e.g., “interbody fusion”, “pedicle screw fixation”, “disc degeneration”) with corresponding image regions, while also preserving the global clinical context necessary for downstream tasks such as classification and segmentation.

### Multimodal global–local representation learning for lumbar fusion analysis

**Fig. 1:**
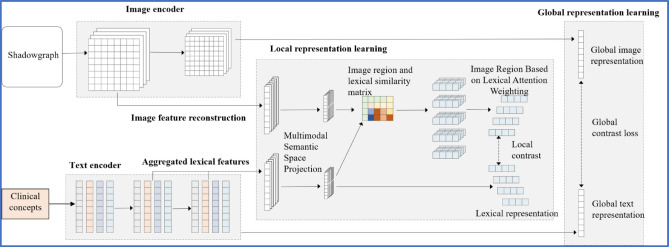
Multimodal clinical assessment system architecture.

To effectively integrate visual and textual information in lumbar fusion surgery, we propose a unified multimodal representation learning framework that jointly models both global anatomical context and fine-grained local correspondences between spinal images and clinical reports. Given a paired sample ($\:{\mathrm{x}}_{\mathrm{v}}$, $\:{\mathrm{x}}_{\mathrm{t}}$), we first extract visual and textual features using the encoders described in Sect. 2.1. These features are subsequently projected into a shared multimodal embedding space through learnable transformation functions. Specifically, global representations $\:{\mathrm{v}}_{\mathrm{g}}$ and $\:{\mathrm{t}}_{\mathrm{g}}$ capture the overall anatomical and clinical semantics, while local representations $\:{\mathrm{v}}_{\mathrm{l}}$ and $\:{\mathrm{t}}_{\mathrm{l}}$ encode region-level visual structures and token-level clinical concepts, respectively. The architecture of the multimodal clinical assessment system is shown in Fig. 1.

#### Multimodal embedding projection

To align heterogeneous modalities, we introduce modality-specific projection functions that map both visual and textual features into a common semantic space. The global representations are defined as:

1 $$\:{v}_{g}={R}_{vg}\left({E}_{v}\right({x}_{v}\left)\right)\:,\hspace{1em}{t}_{g}={R}_{tg}\left({E}_{t}\right({x}_{t}\left)\right)$$

where $\:{\mathrm{R}}_{\mathrm{vg}}$ and $\:{\mathrm{R}}_{\mathrm{tg}}$ are learnable projection functions for image and text modalities.

Similarly, local representations are projected as:

2 $$\:{v}_{l}={R}_{vl}\left({E}_{v}\right({x}_{v}\left)\right)\:,\hspace{1em}{t}_{l}={R}_{tl}\left({E}_{t}\right({x}_{t}\left)\right)$$

where $\:{v}_{l}\in\:{\mathbb{R}}^{D\times\:M}$ encodes ***M*** spatial regions in lumbar images (e.g., vertebrae, discs, implants), and $\:{t}_{l}\in\:{\mathbb{R}}^{D\times\:\mathrm{W}}$ represents clinically meaningful textual units.

To ensure clinically interpretable alignment, token-level embeddings are aggregated into word-level representations, allowing direct correspondence between medical terms (e.g., “fusion segment”, “instrumentation”, “degeneration”) and anatomical regions.

#### Global cross-modal alignment

At the global level, we enforce semantic consistency between the entire lumbar image and its corresponding clinical report. This is achieved through a contrastive learning objective that encourages matched image-text pairs to be closer in the embedding space than mismatched pairs. The global alignment captures holistic clinical understanding, such as overall surgical outcome, spinal curvature, and presence of pathology. This coarse-level supervision provides robust semantic grounding for downstream tasks. The global objective is formulated as minimizing the negative log posterior probability^28^:

3 $$\:L_{g}^{{(v|t)}} = \sum {\:_{{i = 1}}^{N} } 0\log \left( {\frac{{\exp (\left\langle {v_{{gi}} } \right.,\left. {t_{{gi}} } \right\rangle /t_{1} )}}{{\sum {\:_{{k = 1}}^{N} } \exp (\left\langle {v_{{gi}} } \right.,\left. {t_{{gk}} } \right\rangle /t_{1} )}}} \right)$$

where $\:T_{1}$ ∈ R is a scaling temperature parameter, and ⟨$\:{v}_{gi}$, $\:{t}_{gi}$⟩ represents the cosine similarity between the global image representation $\:{v}_{gi}$ and global text features $\:{t}_{gi}$.

Then posterior probability loss for images given text is:

4 $$\:L_{g}^{{(t|v)}} = \sum {\:_{{i = 1}}^{N} } 0\log \left( {\frac{{\exp (\left\langle {v_{{gi}} } \right.,\left. {t_{{gi}} } \right\rangle /t_{1} )}}{{\sum {\:_{{k = 1}}^{N} } \exp (\left\langle {v_{{gk}} } \right.,\left. {t_{{gi}} } \right\rangle /t_{1} )}}} \right)$$

#### Text-guided local region alignment

While global representations capture overall context, lumbar fusion analysis requires precise localization of clinically relevant structures. To this end, we introduce a text-guided attention mechanism that establishes fine-grained correspondences between image regions and clinical terms. We compute similarity scores between local visual features and textual embeddings to generate a relevance map:

5 $$\:s = v_{l}^{T} t_{l} \in R^{{M \times W}}$$

where each element reflects the association between a spinal image region and a clinical concept.

For each textual unit, we derive an attention distribution over image regions, highlighting anatomically relevant locations (e.g., fusion levels, screw positions). The resulting attention-weighted visual representation is defined as a context-aware aggregation of regional features. This mechanism enables the model to focus on clinically significant regions guided by textual semantics, effectively bridging the gap between descriptive reports and spatial anatomical structures.

For each word, attention weights $\:{\mathrm{a}}_{\mathrm{ij}}$ are calculated based on similarity between the word and subregions of images. For the *i-th* word, its attention weights $\:{\mathrm{a}}_{\mathrm{ij}}$ across ***M*** image regions are determined as follows:

6 $$\:a_{{ij}} = \frac{{\exp (s_{{ij}} /t_{2} )}}{{\sum {\:_{{k = 1}}^{M} } \exp (s_{{ik}} /t_{2} )}}$$

Based on attention weights $\:{\mathrm{a}}_{\mathrm{ij}}$, the local image representations are weighted and summed to obtain context-aware image representations $\:{\mathrm{c}}_{\mathrm{i}}$ corresponding to each word, as shown in the following formula:

7 $$\:c_{i} = \sum {\:_{{j = 0}}^{M} } a_{{ij}} v_{j}$$

#### Local contrastive learning

To further enhance fine-grained alignment, a local contrastive objective is introduced that maximizes the similarity between attention-weighted image features and their corresponding textual representations. This objective enforces consistency between specific spinal regions and corresponding clinical descriptions, while simultaneously distinguishing them from unrelated regions and descriptions.

8 $$\:Z(x_{t} ,x_{v} ) = \log \left( {\sum {\:_{{i = 1}}^{W} } \exp \left( {\left\langle {c_{i} } \right.,\left. {t_{i} } \right\rangle /t_{3} } \right)} \right)^{{t_{3} }}$$

Such fine-grained supervision is particularly beneficial for tasks like segmentation, where precise localization of pathological or surgical regions is critical. The overall training objective combines both global and local alignment. Global objective is to capture holistic semantics, and local objective is to enforce fine-grained correspondence. By jointly optimizing these objectives, the model learns complementary representations that encode both contextual understanding and spatial precision.

The local contrast loss functions are defined as follows:

9 $$\:L_{l}^{{(v|t)}} = \sum {\:_{{i = 1}}^{N} } - \log \left( {\frac{{\exp (Z(x_{{vi}} ,x_{{ti}} )/t_{2} )}}{{\sum {\:_{{k = 1}}^{N} } \exp (Z(x_{{vi}} ,x_{{tk}} )/t_{2} )}}} \right)$$

10 $$\:L_{l}^{{(t|v)}} = \sum {\:_{{i = 1}}^{N} } - \log \left( {\frac{{\exp (Z(x_{{vi}} ,x_{{ti}} )/t_{2} )}}{{\sum {\:_{{k = 1}}^{N} } \exp (Z(x_{{vk}} ,x_{{ti}} )/t_{2} )}}} \right)$$

This unified representation is highly effective for downstream lumbar fusion analysis tasks, including: anatomical structure segmentation, surgical outcome classification, and image-text retrieval for clinical decision support. The contrastive learning strategy adopted in this study is bidirectional, including both image-to-text and text-to-image alignment objectives. This symmetric formulation ensures consistent optimization of cross-modal representations in both directions.

The total loss function is as follows:

11 $$\:L = L_{g}^{{(t|v)}} + L_{g}^{{(v|t)}} + L_{l}^{{(t|v)}} + L_{l}^{{(v|t)}}$$

Here, *L*_*g*_ and *L*_*l*_ are defined as the aggregation of bidirectional contrastive losses. Specifically, *L*_*g*_ consists of image-to-text and text-to-image global alignment terms, while *L*_*l*_ includes corresponding local alignment components as defined in Eqs. (9) and (10). Then the total loss is defined as a weighted combination of global alignment, local alignment, and segmentation losses: *L* = *λ*_1_*L*_*global*_ + *λ*_2_*L*_*local*_ + *λ*_3_*L*_*seg*_. To balance the contribution of different components, weighting coefficients are introduced for each loss term. In this study, *λ*_1_, *λ*_2_, and *λ*_3_ are empirically set to 1.0, as the magnitudes of the loss terms are observed to be comparable during training. In addition, we monitor the training dynamics to ensure that no single loss term dominates the optimization process. Experimental results show stable convergence under this configuration.

### Multimodal segmentation framework for lumbar fusion surgery

Building upon the learned global–local multimodal representations, this study further develops a text-guided segmentation framework tailored for lumbar fusion surgery. The goal is to accurately delineate anatomically and clinically relevant structures, such as vertebrae, intervertebral discs, and fusion regions, by leveraging both imaging data and associated clinical descriptions. Unlike conventional segmentation models that rely solely on visual features, our approach incorporates clinical semantics as an additional supervisory signal, enabling more precise localization of surgical regions and pathological structures.

#### Visual encoder initialization

This paper initializes the segmentation backbone using the pretrained visual encoder introduced in Sect. 2.1. This encoder has already learned rich anatomical representations through multimodal alignment, providing a strong initialization for downstream segmentation tasks. The encoded feature maps preserve both: global structural information (spinal curvature, alignment) and local anatomical details (vertebral boundaries, fusion interfaces). These features are then fed into a decoder network for dense prediction.

#### Text-guided feature modulation

**Fig. 2:**
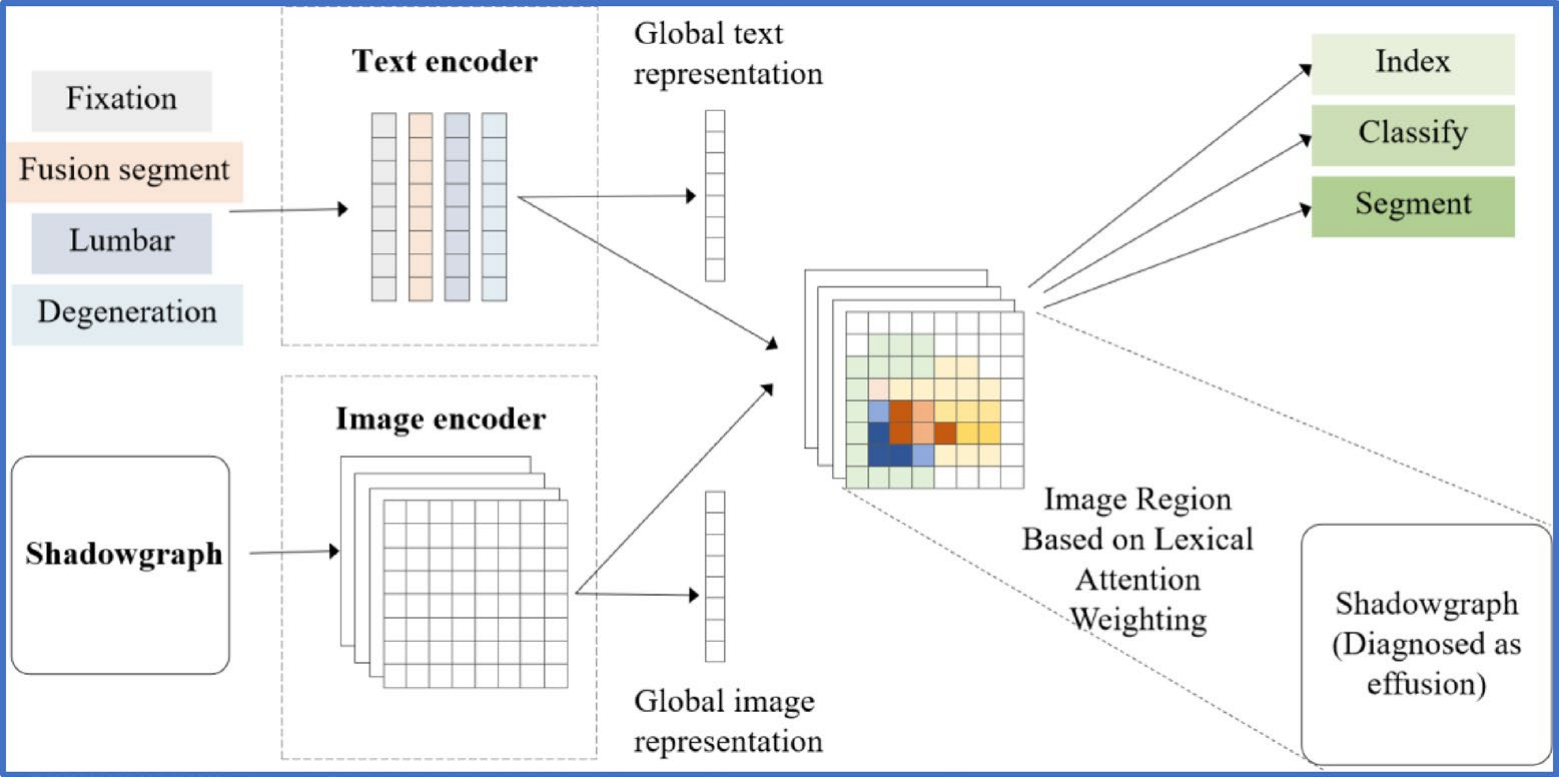
Cross-modal feature alignment mechanism.

To incorporate clinical knowledge into the segmentation process, we introduce a text-guided feature modulation module. Given the textual embeddings extracted from the clinical encoder, we generate semantic guidance signals that interact with visual feature maps through cross-modal attention. Specifically, textual features act as queries representing clinical concepts (e.g., “fusion segment”, “fixation”, “degeneration”), and visual features serve as keys and values, encoding spatial information (see in Fig. 2). This interaction produces attention maps that highlight regions in the image corresponding to clinically relevant terms.

The resulting modulated feature maps enhance the representation of target anatomical structures, especially in challenging cases where visual cues are subtle or ambiguous.

#### Cross-modal attention decoder

It adopts a decoder architecture (UNet-style) augmented with cross-modal attention mechanisms. At each decoding stage, visual feature maps are progressively upsampled, and text-guided attention is applied to refine spatial features.

This design allows the model to suppress irrelevant background regions and emphasize clinically meaningful structures, Furthermore, skip connections from the encoder ensure that fine-grained spatial details are preserved throughout the decoding process.

#### Segmentation objective

The model is trained using a combination of segmentation losses, including dice loss for overlap maximization and cross-entropy loss for pixel-wise classification. Optionally, this research incorporates an auxiliary alignment loss to maintain consistency between segmentation outputs and multimodal representations learned in Sect. 2.2. This multi-objective optimization encourages both accurate boundary delineation and semantic consistency with clinical descriptions.

#### Inference and clinical interpretation

During inference, the model takes a lumbar image as input and optionally incorporates predefined or generated clinical prompts to guide segmentation. The final output is a pixel-wise segmentation map highlighting vertebral bodies, intervertebral discs, fusion regions and surgical implants.

Additionally, the attention maps provide interpretability by revealing which image regions are associated with specific clinical concepts, offering valuable insights for clinical decision support.

#### Structure-aware modeling of lumbar anatomy

**Fig. 3:**
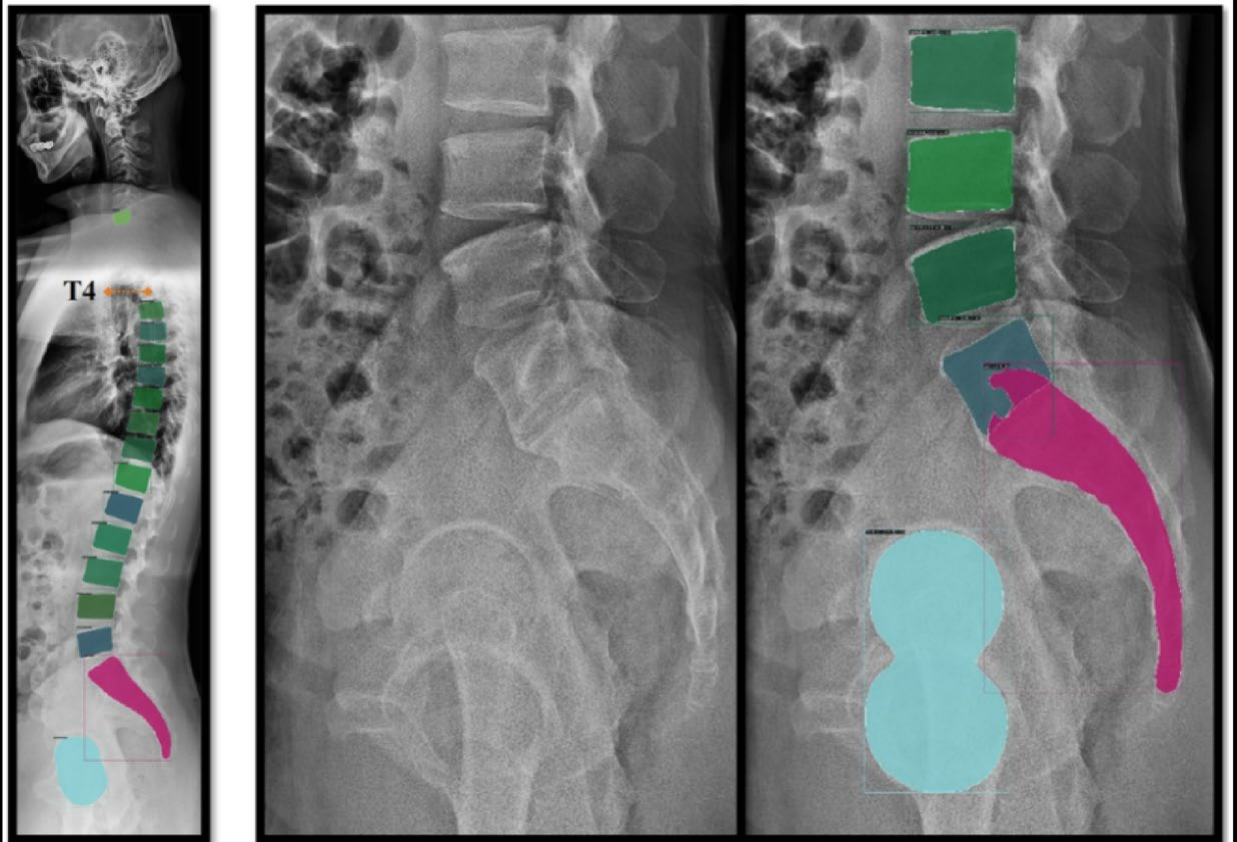
Structure-aware modeling of lumbar anatomical regions. The left panel illustrates the global spinal structure with segmented vertebral regions. The middle panel shows a representative lumbar radiograph. The right panel presents the decomposition of the image into anatomically meaningful components, including vertebral bodies, spinal curvature, and pelvic structures. This structural representation provides prior knowledge to guide multimodal representation learning and improve spatial consistency.

To enhance the representation capability of the proposed framework, anatomical prior knowledge is incorporated through structure-aware modeling of the lumbar spine. Rather than treating the input image as an unstructured collection of pixels, the lumbar region is represented as an organized system of anatomically meaningful components. As illustrated in Fig. 3, the lumbar spine can be decomposed into several structural elements, including vertebral bodies, intervertebral regions, spinal curvature, and surrounding pelvic structures. This decomposition enables the model to explicitly capture spatial relationships and hierarchical organization within the anatomy.

### Implementation and architectural specification

To enhance reproducibility and provide a concrete realization of the proposed framework, we further specify the network architecture and implementation details in this section. The following description corresponds to the theoretical formulation presented in the previous subsections.

#### Backbone architecture

The visual encoder is implemented using a ResNet-50 backbone pretrained on ImageNet. Given an input image $\:X\in\:{\mathbb{R}}^{H\times\:W}$, the encoder produces a feature map $\:{F}_{v}\in\:{\mathbb{R}}^{C\times\:{H}^{{\prime\:}}\times\:{W}^{{\prime\:}}}$, where *C* = 2048 and *H’* = H/32, *W’* = W/32.

The text encoder is implemented using BioClinicalBERT, which generates contextual embeddings $\:{F}_{t}\in\:{\mathbb{R}}^{n\times\:d}$, where *d* = 768 and *n* is the number of tokens. The [CLS] token embedding is used as the global text representation.

#### Global and local feature representation

Global visual features are obtained via global average pooling of *g*_*v*_∈$\:{\mathbb{R}}^{C}$, while local visual features are defined as spatial feature vectors of *L*_*v*_={*f*_*i*_∈$\:{\mathbb{R}}^{C}$} for *i* = 1,…, *H’*×*W’.* Similarly, the global textual feature is *g*_*t*_∈$\:{\mathbb{R}}^{d}$ and local textual features are defined as *L*_*t*_={*t*_*j*_∈$\:{\mathbb{R}}^{d}$} for *j* = 1,…,n.

These representations are used in the global–local alignment formulation defined in Eqs. (9)–(11).

#### Segmentation decoder

The segmentation module is implemented using a U-Net architecture. The encoder weights are initialized from the pretrained visual encoder, while the decoder consists of a series of upsampling and convolutional layers.

Specifically, feature maps are progressively upsampled using bilinear interpolation followed by convolution, and skip connections are applied to fuse encoder and decoder features of *F*_*dec*_=Concat(*F*_*enc*_, *F*_*up*_). The final segmentation output is *S*∈$\:\mathbb{R}$^H×W×K^, where *K* denotes the number of anatomical classes (vertebrae, intervertebral discs, instrumentation). A softmax activation is applied to obtain pixel-wise class probabilities.

#### Loss function clarification

The total loss defined in Eq. (11) is implemented as a weighted combination of global alignment loss, local alignment loss, and segmentation loss *L* = λ_1_*L*_*global*_ + λ_2_*L*_*local*_ + λ_3_*L*_*seg*_, where λ_1_ = 1.0, λ_2_ = 1.0, and λ_3_ = 1.0. The segmentation loss is defined as the sum of Dice loss and cross-entropy loss to balance region overlap and pixel-wise classification.

#### Training strategy

The model is trained using the Adam optimizer with an initial learning rate of 1 × 10^− 4^. The batch size is set to 16. The temperature parameter in contrastive learning is fixed at τ = 0.07.

All models are trained for 100 epochs with standard data augmentation, including rotation, flipping, and intensity perturbation. The implementation is conducted in PyTorch on NVIDIA GPUs.

These implementation details ensure that the proposed framework is fully reproducible and can be consistently evaluated across different experimental settings. The baseline methods include image-only encoders, U-Net-based segmentation models, and CLIP-style multimodal alignment models. For image-only baselines, the same visual encoder architecture is used without textual input. For CLIP-style models, only global image–text alignment is applied without local alignment. For segmentation baselines, standard U-Net architectures are trained using the same input data and annotation settings. All baseline models are reimplemented in our experimental framework to ensure consistency in training conditions and evaluation protocols.

## Results

Based on the proposed framework described in Sect. 2, this section presents experimental results across multiple tasks, including cross-modal retrieval, classification, and segmentation. These tasks are designed to comprehensively evaluate the effectiveness of the learned multimodal representations under a unified dataset and experimental pipeline. To ensure a fair comparison, all baseline methods are implemented and evaluated under the same experimental settings. Specifically, all models are trained using the same dataset splits, identical preprocessing steps, and consistent training protocols. Hyperparameters are either kept consistent across methods or tuned individually within reasonable ranges to achieve optimal performance. All experiments are conducted on the same training, validation, and testing sets defined in Sect. 2, ensuring that performance differences are attributable to model design rather than data variation.

The experiments in this study are conducted on two types of medical imaging data: lumbar spine X-ray images and CT volumes. All datasets are processed under a unified pipeline to ensure consistency across different tasks. The lumbar X-ray dataset consists of 1248 images collected from 312 patients undergoing lumbar spine examination and postoperative follow-up. The dataset includes both anteroposterior and lateral views. Pixel-level annotations were manually provided by experienced radiologists, covering three primary categories: (1) vertebral bodies (L1–L5), (2) intervertebral disc regions, and (3) surgical instrumentation (e.g., pedicle screws and connecting rods). In addition, image-level labels are assigned for classification tasks, including degeneration status, fusion condition, and presence of instrumentation. The CT dataset is based on the publicly available VerSe dataset. In this study, a subset of 185 CT volumes focusing on lumbar regions (L1–L5) is used. Each volume includes voxel-level annotations for vertebral structures, which are used for segmentation evaluation.

For multimodal learning, each image is paired with corresponding clinical text derived from structured reports, including descriptions of vertebral alignment, degeneration status, and postoperative conditions. The pairing is constructed based on examination records to ensure semantic consistency. All images are resized to a fixed resolution and normalized to zero mean and unit variance. Data augmentation techniques, including rotation (± 10°), horizontal flipping, and intensity variation, are applied during training. For CT data, 2D slices are extracted along the sagittal plane for consistency with X-ray-based learning. The dataset is divided into training, validation, and testing sets with a ratio of 70%/10%/20%. The split is performed at the patient level to prevent data leakage and ensure fair evaluation across all tasks.

### Multimodal representation evaluation via cross-modal retrieval

All experiments in this section are conducted on the paired lumbar X-ray dataset (1248 images with corresponding clinical text), as described in Sect. 2. The task is formulated as cross-modal retrieval, where each image is matched against all candidate reports in the test set. To assess the quality of the learned multimodal representations, a cross-modal retrieval task is conducted using the lumbar spine X-ray dataset with paired clinical reports. Given an input image, the goal is to retrieve the most relevant clinical descriptions by measuring the similarity between the image representation and candidate text representations in the shared embedding space. Specifically, each query image is compared against all candidate reports, and similarity scores are computed based on the learned global and local representations. Retrieval performance is evaluated using Precision@K metrics, which measure the proportion of correctly matched reports among top-K retrieved results. The results are summarized in Table 1. When only global representations are used, the model achieves competitive performance compared to image-only alignment methods. This is expected, as global features capture the overall anatomical and clinical semantics of lumbar structures. Using only local representations leads to improved fine-grained matching, indicating that localized features provide more precise correspondence between anatomical regions and clinical terms. The best performance is achieved when both global and local representations are jointly utilized. This combination significantly improves retrieval accuracy across all evaluation metrics, outperforming all baseline methods. The results demonstrate that global and local features provide complementary information, enabling more effective alignment between lumbar images and clinical descriptions.

**Table 1:** Cross-modal retrieval results on the lumbar spine dataset. (All results are evaluated on the lumbar X-ray dataset using the test split defined in Sect. 2).

Method	Prec@5	Prec@10	Prec@100
Image-only baseline	52.31	48.76	36.42
CLIP-style alignment	60.84	57.12	44.65
Ours (global only)	64.25	61.33	47.90
Ours (local only)	66.18	63.05	49.12
Ours (global + local)	69.87	66.54	53.26

### Representation generalization for lumbar diagnosis

The learned multimodal representations are further evaluated on lumbar image classification tasks under two settings: supervised classification and zero-shot classification. For supervised classification, a linear classifier is trained on top of the pretrained visual encoder using different proportions of training data (1%, 10%, and 100%) to assess data efficiency. For zero-shot classification, the approach described in Section 2.3 is applied to predict class labels based on image–text similarity without additional fine-tuning. The classification task is defined as image-level prediction based on annotated labels, including degeneration status, fusion condition, and instrumentation presence. All experiments are conducted on the lumbar X-ray dataset using the predefined patient-level split described in Section 2. Classification performance is evaluated on lumbar X-ray datasets with labels corresponding to anatomical conditions and surgical outcomes (e.g., degeneration, fusion status, instrumentation presence). To reduce variance, results are averaged over multiple runs. The area under the ROC curve (AUROC) is adopted as the primary evaluation metric. The results are presented in Table 2. The proposed method consistently outperforms baseline approaches across all training settings. Notably, models trained with limited data achieve competitive or superior performance compared to fully supervised models initialized with ImageNet weights. This demonstrates that joint global–local representation learning improves feature quality and enhances data efficiency. Although existing multimodal methods adapted from natural image tasks show reasonable performance, their effectiveness is limited in medical imaging scenarios due to high inter-class similarity and subtle anatomical variations. In contrast, the proposed framework learns more discriminative representations by aligning clinically meaningful textual descriptions with anatomical structures. Zero-shot classification results are summarized in Table 3. The model achieves competitive performance without task-specific training labels, indicating strong generalization capability. In particular, the zero-shot setting demonstrates robust performance in identifying lumbar conditions and surgical features, highlighting the effectiveness of multimodal alignment for clinical interpretation tasks. Table 2. Supervised classification results (AUROC) on lumbar datasets. (Classification results are evaluated on the lumbar X-ray dataset with image-level labels)

**Table 2:** Supervised classification results (AUROC) on lumbar datasets. (Classification results are evaluated on the lumbar X-ray dataset with image-level labels).

Method	1%	10%	100%
Random initialization	61.2	68.5	72.3
ImageNet pretrained	75.8	81.6	84.2
UNet encoder	77.4	82.9	85.1
TransUNet encoder	80.6	84.7	86.3
Ours (multimodal)	84.9	87.2	89.1

**Table 3:** Zero-shot classification results on lumbar datasets. (Classification results are evaluated on the lumbar X-ray dataset with image-level labels).

Setting	Acc	Sens	Spec	PPV	NPV	F1
100% supervised	0.81	0.85	0.79	0.66	0.93	0.74
10% supervised	0.78	0.81	0.77	0.63	0.91	0.71
1% supervised	0.72	0.76	0.73	0.58	0.89	0.66
Zero-shot (ours)	0.79	0.83	0.78	0.65	0.92	0.73

### Text-guided lumbar structure segmentation performance

The segmentation task is evaluated on both the lumbar X-ray dataset (pixel-level annotations) and the VerSe CT dataset (voxel-level annotations). For X-ray images, segmentation targets include vertebral bodies, intervertebral disc regions, and instrumentation. For CT data, segmentation focuses on vertebral structures (L1–L5). The effectiveness of the proposed multimodal representation framework is further evaluated on lumbar structure segmentation tasks. A UNet-based architecture is adopted, where the encoder is initialized using the pretrained visual encoder. This initialization enables the model to leverage both anatomical and clinical semantic information learned from multimodal alignment. Performance is compared against different initialization strategies, including random initialization, ImageNet pretraining, and image-only models without textual guidance. Segmentation accuracy is evaluated using the Dice coefficient under varying amounts of training data (1%, 10%, and 100%), in order to assess data efficiency. The results are summarized in Table 4. The proposed method consistently outperforms all baselines across different training settings. In particular, substantial improvements are observed under low-data regimes, where the availability of segmentation annotations is limited. This indicates that multimodal representation learning effectively enhances feature quality and reduces reliance on large amounts of labeled data. To further evaluate the contribution of local alignment, an additional ablation experiment using CLIP-style initialization (global alignment only) is included. As shown in Table 4, the global-only model achieves improved performance compared to image-only baselines, indicating that cross-modal global alignment provides useful semantic guidance. However, the proposed method with both global and local alignment further improves segmentation performance across all training settings. This demonstrates that local alignment contributes additional fine-grained structural information, which is particularly beneficial for precise anatomical segmentation. It should be noted that attention visualization provides qualitative insights into the model’s focus but does not constitute a definitive explanation of model decision-making. The observed attention patterns should be interpreted with caution, as they may not fully reflect causal relationships between input features and model outputs.

**Table 4:** Lumbar structure segmentation results (Dice score) on X-ray and VerSe CT datasets. (The results are evaluated using the same training/testing split across all methods).

Initialization method	1%	10%	100%
Random	0.58	0.71	0.82
ImageNet	0.62	0.75	0.85
Image-only (encoder)	0.68	0.79	0.87
CLIP-style (global only)	0.70	0.81	0.87
Ours (multimodal, global+local)	0.74	0.83	0.89

### Clinical interpretability via attention visualization

**Fig. 4:**
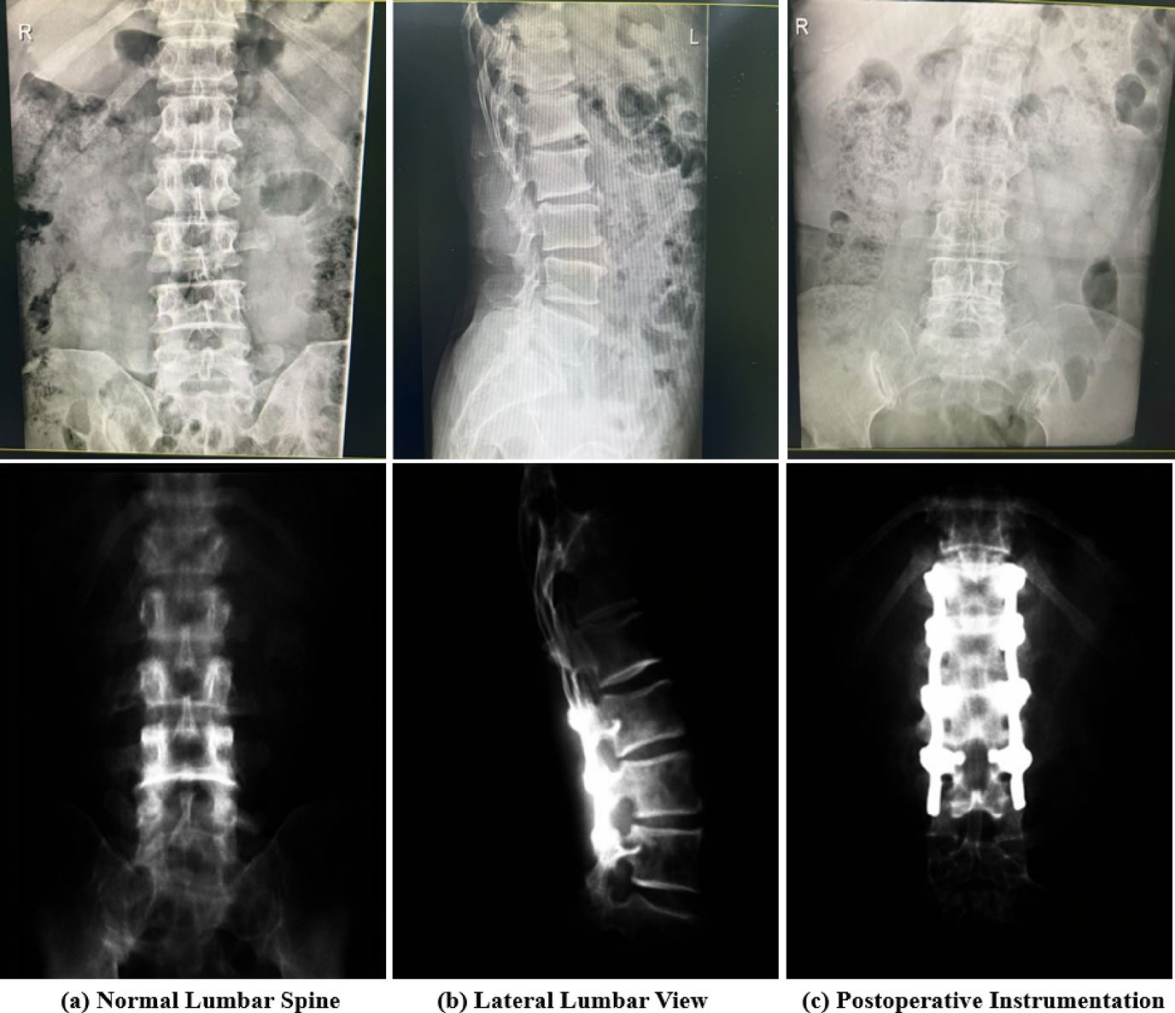
Visualization of text-guided attention on frontal lumbar radiographs. The top row shows input images, while the bottom row illustrates attention maps highlighting clinically relevant anatomical regions associated with specific textual concepts.

To qualitatively evaluate the interpretability of the proposed framework, attention maps derived from the text-guided alignment mechanism are visualized. These attention weights highlight image regions that are most relevant to specific clinical concepts, providing insight into how textual information guides spatial localization. The attention maps are resized to match the input image resolution and overlaid on the original lumbar X-ray images. As shown in Fig. 4, the model effectively identifies clinically meaningful regions corresponding to different anatomical and surgical concepts. For example, attention guided by the term “fusion segment” highlights the intervertebral region where fusion has been performed. Similarly, terms such as“pedicle screw” and “instrumentation” activate regions corresponding to implanted hardware. In cases involving “disc degeneration,” the attention maps focus on narrowed disc spaces, indicating degeneration-related changes. In Fig. 4, the highlighted regions generally correspond to anatomically relevant structures, such as vertebral bodies and instrumentation. However, these visualizations should be considered as qualitative observations rather than rigorous interpretability validation.

**Fig. 5:**
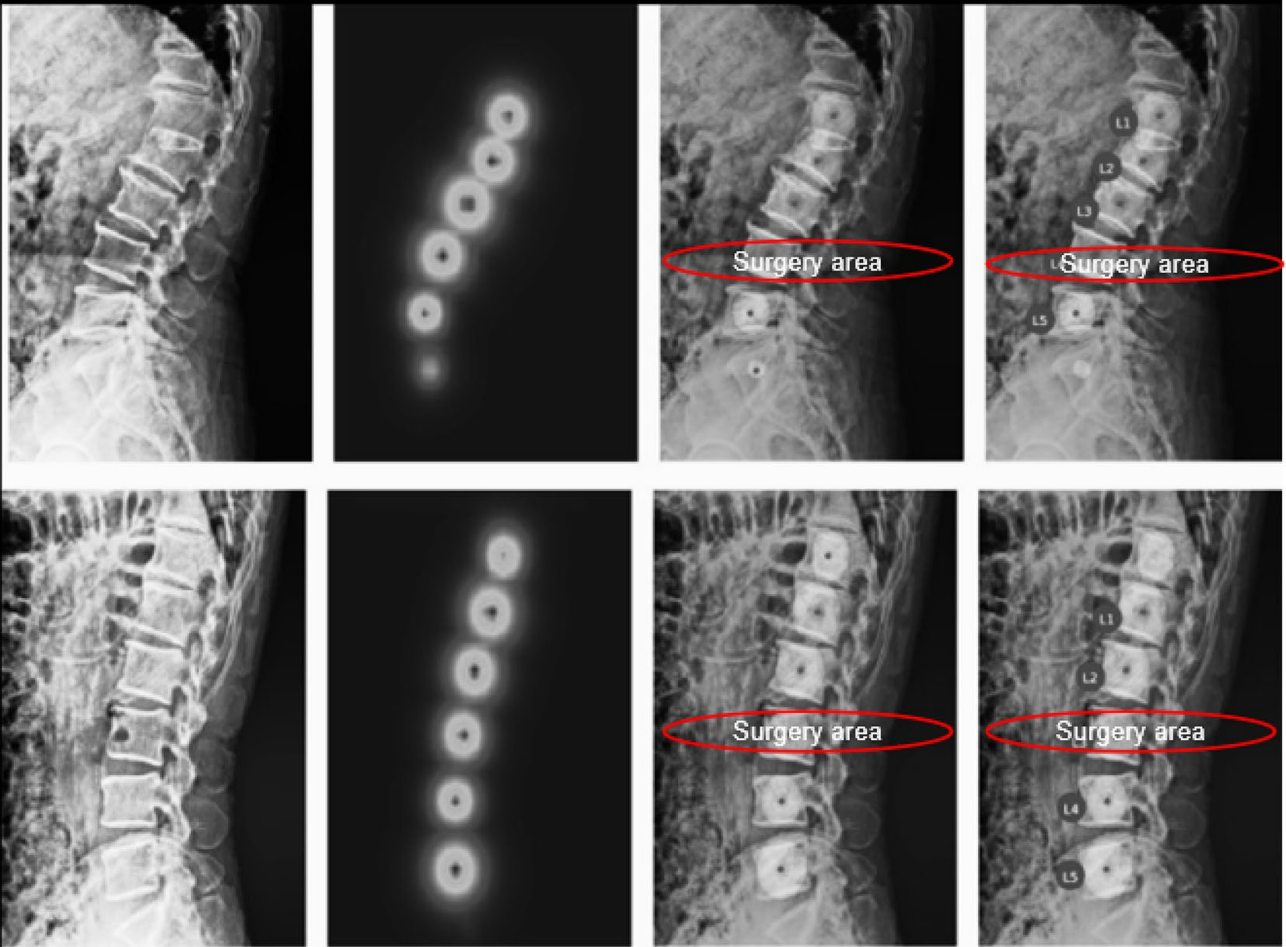
Attention visualization for lumbar vertebra localization and interpretability analysis. The first column shows the input radiographs. The second column presents the corresponding attention heatmaps. The third column illustrates the overlay of attention responses on the original images. The fourth column shows the automatically localized vertebral regions with anatomical labels (L1–L5). The results demonstrate that the model focuses on clinically relevant regions and captures meaningful anatomical structures.

The interpretability of the proposed framework is further analyzed through attention visualization, as shown in Fig. 5. The generated attention maps highlight the regions that contribute most to the model’s decision-making process. It can be observed that the model consistently focuses on anatomically meaningful areas corresponding to the lumbar vertebrae. The attention responses are well aligned with the spatial locations of vertebral bodies, indicating that the model captures relevant structural information rather than relying on irrelevant background features. Furthermore, the automatically localized vertebral regions with corresponding labels (L1–L5) demonstrate that the model is capable of learning hierarchical anatomical relationships. These results provide strong evidence that the proposed method achieves not only accurate predictions but also clinically meaningful interpretability, which is essential for reliable medical image analysis.

It should be noted that attention visualization provides qualitative insights into the model’s focus but does not constitute a definitive explanation of model decision-making. The observed attention patterns should be interpreted with caution, as they may not fully reflect causal relationships between input features and model outputs.

### Structural variation analysis

In addition to modeling anatomical structure, the proposed framework is capable of capturing structural variations in the lumbar spine, which are critical for identifying pathological changes. These variations include alterations in intervertebral spacing, irregular vertebral alignment, and postoperative structural changes. Such structural patterns are closely associated with clinically relevant conditions, including disc degeneration, intervertebral space narrowing, and instability following surgical intervention. By learning region-level representations and spatial relationships, the model effectively encodes these variations without relying on explicit geometric assumptions.

**Fig. 6:**
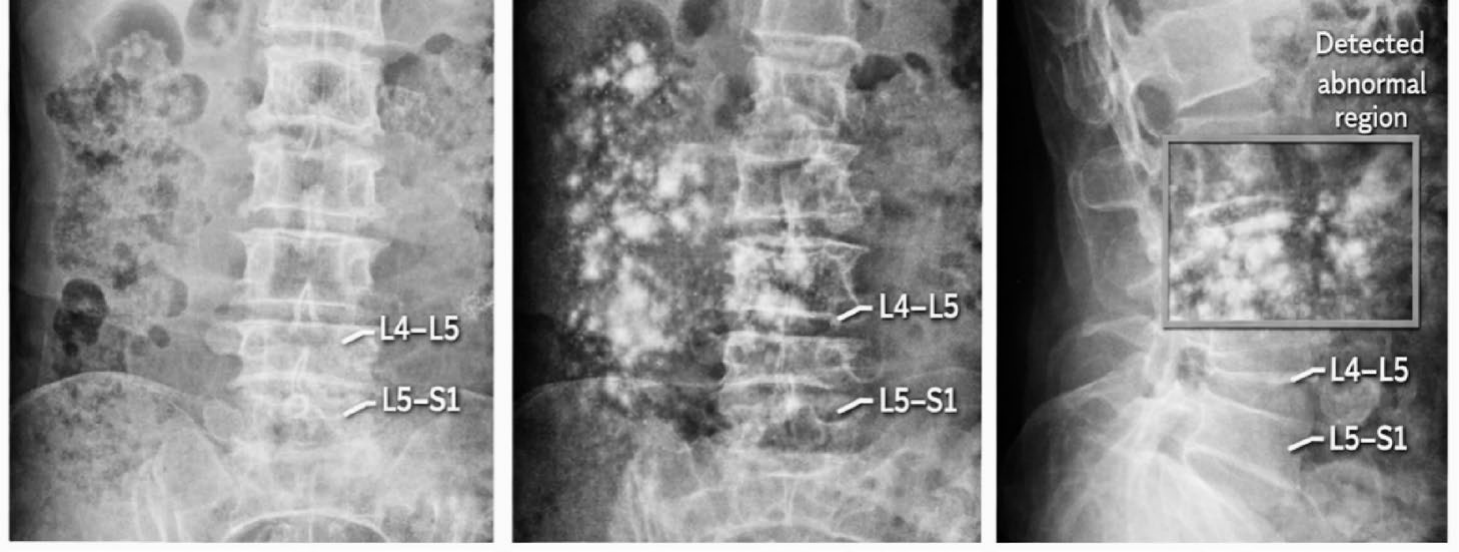
Visualization of structural variations and abnormal region detection in lumbar spine radiographs.

Quantitative evaluation demonstrates that incorporating structure-aware representations improves classification performance in distinguishing normal and abnormal cases. The accuracy increases from 78.5% (baseline) to 86.3%, indicating that structural information provides strong discriminative cues for clinical assessment. Furthermore, visual analysis supports these findings. As illustrated in Fig. 6, the model consistently highlights regions exhibiting significant structural changes, particularly in lower lumbar segments such as L4–L5 and L5–S1. These regions correspond to common sites of degeneration, suggesting that the model captures clinically meaningful anatomical variations.

The stability of the learned representations was evaluated by analyzing their structural consistency across different samples and imaging conditions. The proposed framework demonstrates strong robustness, effectively preserving essential anatomical structures while reducing the influence of noise and irrelevant variations. Quantitatively, the proposed method achieves improved consistency compared with the baseline, indicating that the learned features are more stable and discriminative. This suggests that incorporating structure-aware modeling enhances the reliability of the representations. Visual comparisons further support these findings. The representations generated by the proposed method maintain clearer anatomical boundaries and more coherent structural patterns, especially in regions with complex variations. In contrast, the baseline model exhibits less stable responses and is more affected by local noise. The quantitative comparison is summarized in Table5. The proposed method achieves lower variability and higher structural consistency than the baseline, demonstrating its effectiveness in preserving meaningful anatomical information.

### Clinical data description and application context

**Fig. 7:**
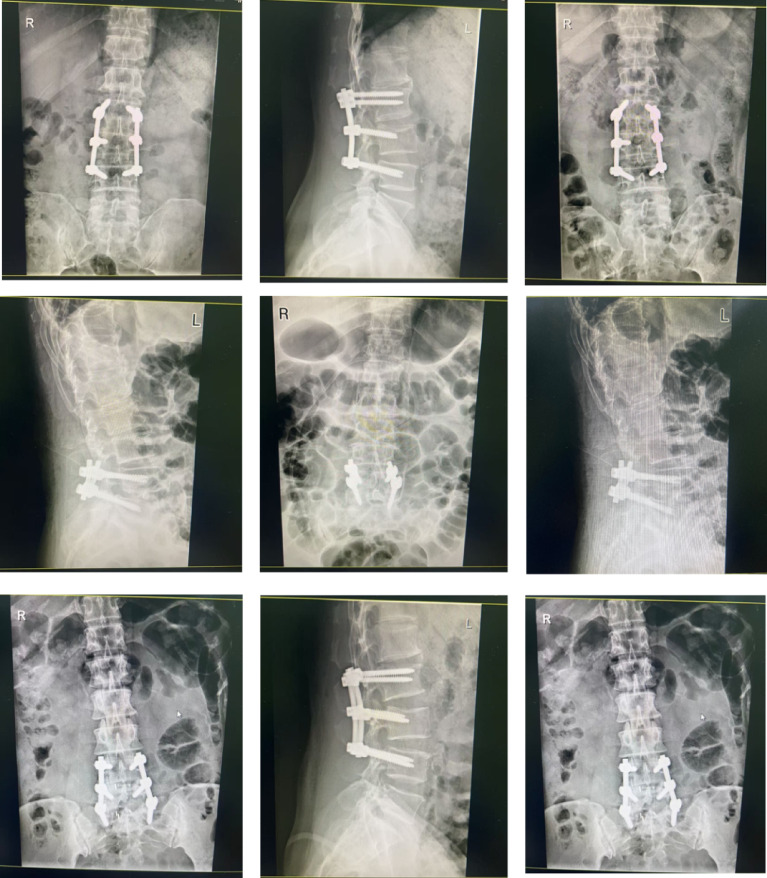
Representative lumbar spine radiographs with postoperative instrumentation.

The images shown in Fig. 7 present a subset of the collected lumbar spine radiographic dataset, including both anteroposterior (AP) and lateral views. These cases illustrate typical postoperative conditions following lumbar fusion surgery, where pedicle screw fixation systems have been implanted to stabilize the spine. The instrumentation structures, including screws and connecting rods, are clearly visible across different imaging perspectives. The dataset covers a variety of clinical scenarios, including multi-level fixation, short-segment stabilization, and unilateral or bilateral screw placements. In addition, variations in anatomical structures, imaging quality, and interference from surrounding tissues (e.g., intestinal gas) are observed, reflecting the complexity of real-world clinical data. Some cases also exhibit degenerative changes such as reduced intervertebral disc space and irregular vertebral alignment, which are common indications for surgical intervention. These images are used as part of the experimental dataset for both training and evaluation of the proposed method. Specifically, they provide diverse examples for learning robust representations of vertebral structures and implanted instrumentation. The variability in imaging conditions and surgical configurations further supports the assessment of the model’s generalization capability in practical clinical settings. This dataset enables comprehensive evaluation of the proposed framework in realistic postoperative imaging scenarios.

**Fig. 8:**
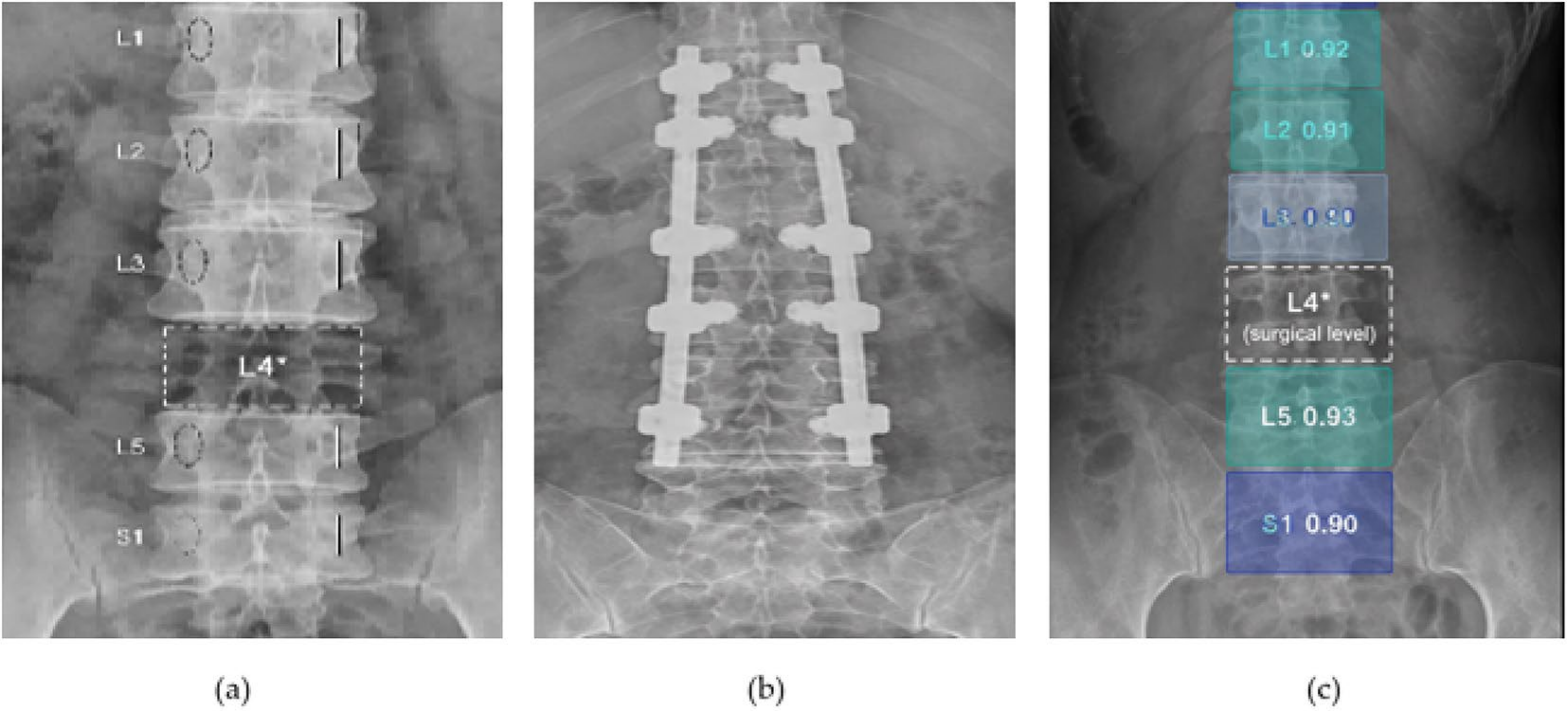
Visualization of lumbar structure analysis and instrumentation detection. (**a**) Anatomical landmark localization. The left image shows the identification of key anatomical landmarks along the lumbar spine. The highlighted regions (circled in red) indicate representative structural points, which are useful for capturing spatial alignment and vertebral positioning. These localized features provide important structural cues for downstream analysis; (**b**) Postoperative instrumentation visualization. The middle images present typical postoperative cases with pedicle screw fixation systems in both anteroposterior and lateral views. The implanted screws and rods are clearly visible, reflecting common surgical interventions for lumbar stabilization. These examples illustrate the structural variability of instrumentation across different patients and imaging perspectives; (**c**) Automated vertebral region detection. The right image demonstrates the automatic detection and localization of lumbar vertebral regions. The model assigns bounding boxes to different vertebral levels with corresponding confidence scores, indicating its ability to identify and differentiate anatomical segments. This step provides structured region-level information for further analysis tasks such as classification or segmentation.

Fig. 8 illustrates representative results of lumbar spine analysis, including anatomical localization, postoperative instrumentation visualization, and automated vertebral region detection. The examples demonstrate the capability of the proposed framework to capture structural features and identify clinically relevant regions from lumbar radiographs under different imaging conditions. These visualizations highlight the effectiveness of the proposed framework in integrating structural feature extraction and region-level localization. The detected anatomical regions and instrumentation features serve as important inputs for subsequent representation learning and segmentation tasks, supporting comprehensive lumbar spine analysis.

Overall, the proposed framework consistently outperforms baseline methods across all evaluation tasks. The results demonstrate that integrating multimodal representation learning with global–local alignment improves both semantic understanding and structural prediction in lumbar spine imaging.

**Fig. 9:**
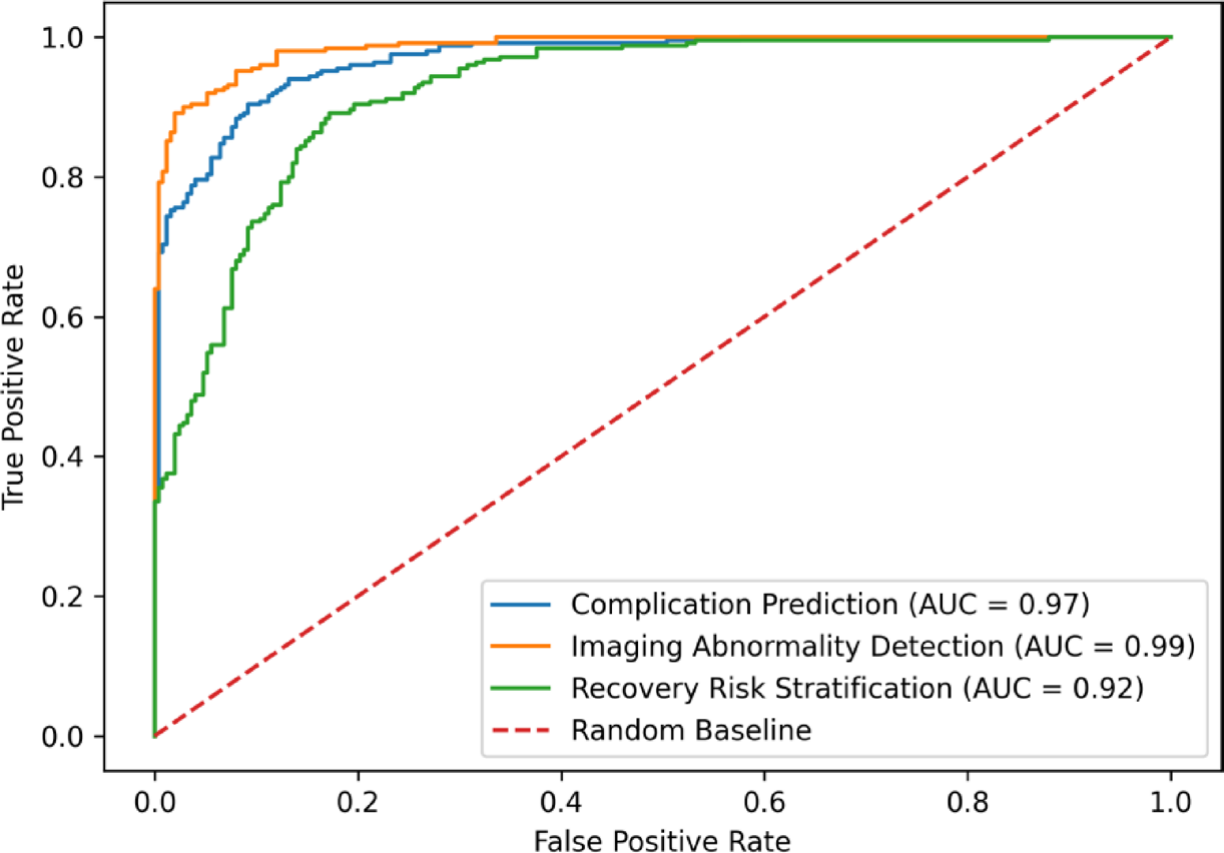
ROC Curves of the Multimodal Clinical Assessment Model.

## Discussion

**Fig. 10:**
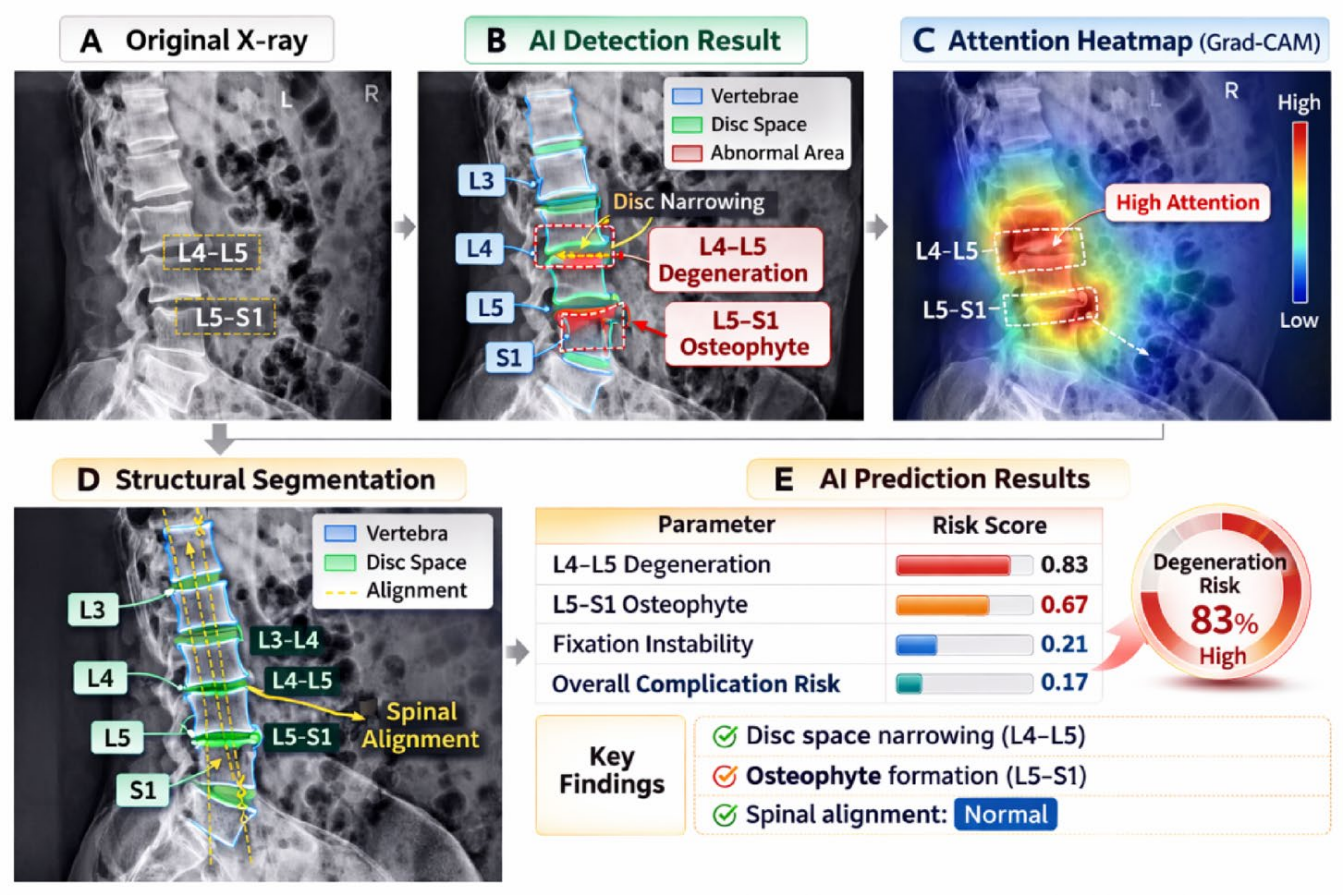
AI-assisted radiographic analysis of lumbar spine X-ray images. (**A**): Original radiographic image. (**B**): AI detection results showing vertebral localization and abnormal regions identified by bounding boxes. (**C**): Attention heatmap visualization highlighting regions with high model attention, particularly around the L4–L5 and L5–S1 segments. (**D**–**E**): Structural segmentation and disc space analysis used for quantitative assessment of degeneration and spinal alignment.)

The cross-modal similarity model was able to effectively associate imaging findings with textual clinical descriptions, enabling more accurate identification of abnormal postoperative patterns. Compared with manual assessment based solely on clinical records, the AI-assisted framework demonstrated improved agreement with clinical assessment. Receiver operating characteristic (ROC) curves illustrating the performance of the multimodal model are shown in Fig. 9. Representative examples of AI-assisted radiographic analysis are shown in Fig. 10. The proposed multimodal framework automatically identified lumbar vertebral structures and highlighted suspicious regions using bounding-box detection and attention heatmap visualization. The model focused primarily on the L4–L5 and L5–S1 intervertebral segments, which corresponded to clinically relevant degenerative changes such as disc space narrowing and osteophyte formation. In addition to structural localization, the model generated quantitative risk scores for different pathological indicators, including degeneration risk, disc space narrowing, and vertebral instability. These visualization results demonstrate that the multimodal analysis framework can effectively capture imaging features associated with postoperative recovery and potential complications, providing interpretable support for clinical assessment.

The experimental results demonstrate that the proposed multimodal framework effectively integrates visual and textual information for medical image interpretation. The global–local alignment mechanism contributes to improved representation quality, while the text-guided attention mechanism enhances anatomical localization. Compared with image-only approaches, the multimodal framework provides richer semantic context, which is particularly beneficial for complex anatomical structures such as the lumbar spine. In addition, the improved segmentation performance indicates that cross-modal learning can support precise structural analysis. Despite these promising results, several limitations remain. First, the dataset size is relatively limited, and further validation on larger and more diverse datasets is required. Second, the interpretability of the model is currently based on attention visualization, which provides qualitative insights but does not constitute a complete explanation of model behavior. Future work will explore more rigorous interpretability methods and extend the framework to additional clinical tasks. Although attention-based visualization provides intuitive insights into model behavior, it does not offer a complete or reliable explanation of the decision-making process. In medical applications, where spurious correlations may exist, more rigorous interpretability methods, such as causal analysis or attribution-based evaluation, are required. This will be explored in future work. A quantitative evaluation of interpretability is beyond the scope of this study and will be investigated in future work.

## Conclusions

This study addresses the problem of multimodal medical image interpretation in lumbar fusion surgery by proposing a unified representation learning framework that integrates medical imaging data with clinical textual information. By combining global semantic alignment with local structural modeling, the proposed framework enables joint learning of anatomical features and clinical context within a consistent optimization framework.

Experimental results across multiple tasks, including cross-modal retrieval, classification, and segmentation, demonstrate that the proposed method consistently outperforms conventional image-only approaches. The integration of global and local representations allows the model to capture both holistic anatomical context and fine-grained structural variations, leading to improved performance in lumbar spine analysis. In addition, the text-guided attention mechanism provides qualitative visualization of model focus, which may help highlight clinically relevant regions. However, such visualizations should be interpreted with caution, as they do not constitute a complete or causal explanation of model decision-making. Compared with existing multimodal approaches adapted from natural image domains, the proposed framework is better suited for medical imaging scenarios, where structural consistency and subtle anatomical variations are critical. The incorporation of structure-aware modeling contributes to more stable representations and improved preservation of spatial relationships.

Despite these promising results, several limitations remain. The current study is evaluated on a relatively limited dataset, and further validation on larger and more diverse clinical data is required. Moreover, more rigorous interpretability evaluation and validation strategies are needed in future work.

Overall, this work demonstrates that multimodal representation learning is a promising direction for medical image interpretation. The proposed framework provides a structured and extensible solution for integrating heterogeneous clinical data, offering potential for future research in computer-aided diagnosis and intelligent medical imaging systems.

**Table 5:** Comparison of representation stability and structural consistency.

Method	Feature variability	Structural consistency	Localization stability
Baseline (Image-only)	0.128 ± 0.021	0.742	0.761
Global-only	0.109 ± 0.018	0.781	0.803
Local-only	0.102 ± 0.017	0.795	0.812
Proposed method	0.087 ± 0.014	0.832	0.846

## Supplementary Information

Below is the link to the electronic supplementary material.

Supplementary Material 1

## Data Availability

All data in this article are provided in the main text and attachments. The de-identified individual participant data underlying this study are available upon reasonable request from the corresponding author (huangxl@srrsh.com) for non-commercial research purposes. Data sharing is subject to approval by the Ethics Committee of Sir Run Run Hospital and compliance with China’s Personal Information Protection Law.
